# Assessment of Maternal–Fetal Redox Balance in Gestational Diabetes Mellitus: A Cross-Sectional Study

**DOI:** 10.3390/jcm14197003

**Published:** 2025-10-03

**Authors:** Sorina Cristina Chelu, Veronica Daniela Chiriac, Diana Andrei, Emil Robert Stoicescu, Claudia Borza

**Affiliations:** 1 Doctoral School, Victor Babes University of Medicine and Pharmacy Timisoara, Eftimie Murgu Square No. 2, 300041 Timisoara, Romania; sorina.chelu@umft.ro; 2 Centre for Translational Research and Systems, Victor Babes University of Medicine and Pharmacy Timisoara, Eftimie Murgu Square No. 2, 300041 Timisoara, Romania; borza.claudia@umft.ro; 3 Clinic of Obstetrics and Gynecology, Timisoara City Hospital, Hector 2A, 300041 Timisoara, Romania; 4 Discipline of Obstetrics and Gynecology, Victor Babes University of Medicine and Pharmacy Timisoara, Eftimie Murgu Square No. 2, 300041 Timisoara, Romania; 5 Department of Balneology, Medical Rehabilitation and Rheumatology, Victor Babes University of Medicine and Pharmacy Timisoara, Eftimie Murgu Square No. 2, 300041 Timisoara, Romania; 6 Radiology and Medical Imaging University Clinic, Victor Babes University of Medicine and Pharmacy Timisoara, Eftimie Murgu Square No. 2, 300041 Timisoara, Romania; stoicescu.emil@umft.ro; 7 Research Center for Pharmaco-Toxicological Evaluations, Victor Babes University of Medicine and Pharmacy Timisoara, Eftimie Murgu Square No. 2, 300041 Timisoara, Romania; 8 Field of Applied Engineering Sciences, Specialization Statistical Methods and Techniques in Health and Clinical Research, Faculty of Mechanics, Politehnica University Timisoara, Mihai Viteazul Boulevard No. 1, 300222 Timisoara, Romania; 9 Research Center for Medical Communication, Victor Babes University of Medicine and Pharmacy Timisoara, Eftimie Murgu Square No. 2, 300041 Timisoara, Romania; 10 Department of Functional Sciences, Discipline of Pathophysiology, Victor Babes University of Medicine and Pharmacy Timisoara, Eftimie Murgu Square No. 2, 300041 Timisoara, Romania; 11 Centre of Cognitive Research in Pathological Neuro-Psychiatry NEUROPSY-COG, Victor Babes University of Medicine and Pharmacy Timisoara, Eftimie Murgu Square No. 2, 300041 Timisoara, Romania

**Keywords:** gestational diabetes, oxidative stress, cord blood, antioxidant defense, reactive oxygen metabolites, biological antioxidant potential

## Abstract

**Background/Objectives**: Gestational diabetes mellitus (GDM) is one of the most common metabolic complications of pregnancy and is linked to long-term metabolic and cardiovascular risks for both mother and child. Its pathophysiology includes increased generation of reactive oxygen species (ROS) and/or decreased antioxidant defenses; nonetheless, the redox dynamics between mother and fetus are still poorly understood. Our goal was to assess oxidative stress (via derivatives of reactive oxygen metabolites, d-ROMs) and antioxidant capacity (via biological antioxidant potential, BAP) in maternal, umbilical cord, and neonatal blood from women with GDM compared to normoglycemic controls, and to investigate potential associations with clinical and neonatal outcomes. **Methods**: In this single-center cross-sectional study, 56 women with GDM and 52 matched controls provided maternal venous, umbilical cord, and neonatal blood samples at delivery. Plasma d-ROMs and BAP were measured using colorimetric assays. Clinical and neonatal outcome data were collected. **Results**: Women with GDM had considerably higher maternal d-ROM levels compared to both the umbilical cord and neonatal compartments. BAP measurements revealed that maternal blood had the lowest antioxidant capacity, while cord and newborn samples had higher levels. GDM mothers had significantly greater maternal d-ROMs and lower BAP compared to controls (both *p* < 0.05). There were no differences in cord blood d-ROMs or BAP between the GDM and the control group. The maternal BAP/d-ROM ratio decreased significantly in the GDM group (*p* < 0.01), but the cord ratio remained constant. Notably, neither maternal nor neonatal redox indicators were related to perinatal outcomes, indicating a limited prognostic potential for unfavorable neonatal occurrences. **Conclusions**: GDM is associated with increased maternal oxidative stress and decreased antioxidant capacity, with no substantial changes in newborn redox status. Redox indicators did not predict perinatal issues across this group. These findings demonstrate the need for larger prospective research to determine whether early changes in redox balance can predict the development of GDM or unfavorable outcomes.

## 1. Introduction

Gestational diabetes mellitus (GDM) is defined as any degree of glucose intolerance first recognized during pregnancy, and it typically emerges between 24 and 28 weeks of gestation [1,2,3]. The prevalence of GDM varies widely worldwide, with estimates ranging from 1% to over 30%, largely due to differences in diagnostic criteria and screening strategies. It represents one of the most common high-risk metabolic complications of pregnancy. Both mothers with GDM and their offspring face a substantially elevated long-term risk of type 2 diabetes, metabolic syndrome, and cardiovascular disease [4]. In recent decades, advanced maternal age, shifting dietary patterns, and sedentary lifestyles have driven a marked rise in GDM incidence [5].

Pregnancy is characterized by high energy demands and oxygen consumption by both the placenta and the fetus [6]. It reflects a distinct physiological condition characterized by significant metabolic, hormonal, and circulatory changes. As the placenta develops and maternal organs adapt to the expanding fetus, oxygen demand rises and mitochondrial activity increases in the placenta and in maternal tissues, especially in the uterus, heart, and kidneys [7,8,9]. During this time, both maternal and fetal metabolism generate reactive oxygen species (ROS). ROS are important signaling molecules for processes such as angiogenesis and immunological regulation, but excessive synthesis can cause oxidative damage [10].

Under healthy conditions, the body’s enzymatic (superoxide dismutase, catalase, glutathione peroxidase) and non-enzymatic (vitamins C and E, glutathione, uric acid) antioxidant systems neutralize ROS, maintaining redox homeostasis [11]. In contrast, pathological pregnancies, such as those complicated by GDM, exhibit excessive ROS production and/or deficient antioxidant defenses, tipping the balance toward oxidative injury [12]. Additionally, factors such as maternal obesity, smoking, environmental pollutants, or poor nutrition can overwhelm these defenses, tipping the balance toward oxidative stress [13,14,15,16]. This amplified oxidative stress impairs maternal vascular function, disrupts placental perfusion, and compromises fetal development. Excessive oxidative stress in pregnancy can also damage cellular biomolecules, deoxyribonucleic acid (DNA), proteins, lipids, and carbohydrates, yet the precise biochemical pathways remain complex [17]. In pregnancies complicated by diabetes, neural tube and cardiac malformations are two of the most commonly observed congenital anomalies [18,19]. Multiple pathways link hyperglycemia in GDM to oxidative stress. Excess glucose impairs mitochondrial electron transport, driving superoxide overproduction, while additional ROS arise via the polyol, hexosamine, and protein kinase C (PKC) signaling cascades and during the formation of advanced glycation end-products [20].

In the fetal compartment, redox regulation becomes more complex. While its own growing defenses strive to minimize damage, the fetus’s immature antioxidant capacity must compete with ROS that cross the placenta from the maternal blood [21,22]. Therefore, a thorough understanding of the maternal-fetal redox interaction can be obtained by assessing the prooxidant burden and antioxidant capacity in maternal, umbilical cord, and fetal samples. Given the damaging potential of ROS, organisms have evolved multiple antioxidant defenses to protect tissues. These include both endogenous and exogenous antioxidants that inhibit radical formation, neutralize ROS, and terminate oxidative chain reactions [23].

Our aim was to investigate whether GDM is associated with altered oxidative stress (d-ROMs) levels and antioxidant profiles (BAP) in maternal, cord, and neonatal compartments and to assess how these redox parameters relate to clinical and neonatal outcomes.

## 2. Materials and Methods

### 2.1. Study Design and Participants

This cross-sectional study enrolled pregnant women attending the Municipal Emergency Clinical Hospital in Timisoara, between February 2023 and March 2024. Pregnant women attending routine prenatal care were screened and enrolled during this period. The study included one group consisting of 56 pregnant women diagnosed with GDM and 52 normoglycemic, age-matched controls. All women in both groups underwent elective cesarean section after 37 weeks, which ensured standardized timing and conditions for sample collection. GDM was defined according to standard oral glucose tolerance criteria: fasting plasma glucose ≥ 92 mg/dL, 1 h ≥ 180 mg/dL, or 2 h ≥ 153 mg/dL after a 75 g OGTT. Women with at least one abnormal value were classified as having GDM [24]. The study was conducted in accordance with the Declaration of Helsinki [25] and received ethical approval from the Research Ethics Committee of Timiş County Emergency Clinical Hospital, Timișoara, Romania (approval no. 4756/28.02.2022). All participants provided written informed consent.

The inclusion criteria were as follows: singleton pregnancy, maternal age between 18 and 45 years, gestational age between 24 and 36 weeks at enrollment, and availability of clinical and laboratory data. Women in the GDM group had a diagnosis confirmed by oral glucose tolerance test (OGTT). Exclusion criteria included: pregestational diabetes, multiple gestation, major fetal anomalies, chronic inflammatory diseases, acute infection at the time of sampling, or incomplete clinical records. To minimize selection bias, a consecutive sampling strategy was applied, with all eligible women invited during the study period.

Sample size was estimated based on previous studies comparing oxidative stress markers in GDM and normoglycemic pregnancies. Assuming a mean difference of ~150 Carratelli units in d-ROMs, a standard deviation of 200, α = 0.05, and power = 0.80, the minimum required sample size was ~50 per group. To account for potential dropouts and missing data, the recruitment target was set at 55–60 women per group.

### 2.2. Sample Collection

A consecutive sampling strategy was used, whereby all eligible women presenting during the study period were invited to participate. Maternal venous blood (5 mL) was drawn antepartum, in the third trimester of pregnancy (32–36 weeks). Samples were drawn in the morning after an overnight fast of at least 8 h, using EDTA-coated vacutainer tubes as the anticoagulant. Immediately after collection, tubes were placed on ice and transported to the laboratory within 15 min. Plasma was separated by centrifugation at 1500 g for 10 min at 4 °C, aliquoted, and assayed within 2 h of sampling. Neonatal-compartment blood and umbilical cord blood were collected at the time of delivery. Umbilical cord blood (5 mL) was obtained from the umbilical vein immediately after clamping but before placental expulsion, following standard neonatal sampling protocols, in EDTA tubes. Separately, neonatal blood samples (2 mL) were drawn directly from the neonate immediately after birth Via peripheral venipuncture, within the first hour of life under sterile conditions, again in EDTA tubes, prior to any medical intervention. Both umbilical cord and neonatal samples underwent the same handling as maternal samples (placed on ice, centrifuged within 15 min, and analyzed within 2 h). All samples were collected under sterile conditions by experienced clinical personnel. All samples were processed in duplicate, and specimens that were hemolyzed or lipemic were excluded from analysis to minimize pre-analytical variability. Neonatal blood samples were collected only for the GDM group. This reflects routine clinical practice, as infants of mothers with GDM undergo postnatal glucose monitoring, allowing sampling to be performed without additional invasive procedures. Therefore, neonatal compartment analyses are presented exclusively for infants born to mothers with GDM, whereas maternal and cord blood were obtained in both groups.

### 2.3. Oxidative-Stress and Antioxidant Assays

Reactive oxygen metabolites were quantified using the d-ROMs test (Diacron International, Grosseto, Italy). In this assay, plasma hydroperoxides (ROOH) are cleaved in an acidic buffer in the presence of transition metals to generate alkoxy and peroxy radicals, which then oxidize the chromogen N,N-diethyl-p-phenylenediamine to a stable colored radical cation measurable at 505 nm. Results are reported in Carratelli units (U CARR; 1 U CARR = 0.8 mg/L H_2_O_2_ equivalents). Total antioxidant capacity was assessed by the BAP test (Diacron International, Grosseto, Italy), which is based on the reduction in the ferric (Fe^3+^)–thiocyanate complex to ferrous (Fe^2+^), producing a decrease in absorbance at 505 nm proportional to the antioxidant power of the plasma. Results are expressed in µmol/L Fe^2+^ equivalents. For each sample, we determined d-ROMs and BAP and calculated the BAP/d-ROM ratio. For the d-ROMs test, the intra-assay coefficient of variation (CV) was 2.3%, and the inter-assay CV was 2.0%. For the BAP test, the intra-assay CV was 2.5%, and the inter-assay CV was 3.4%. The analytical sensitivity (limit of detection) was ~10 U CARR (≈8 mg/L H_2_O_2_ equivalents) for the d-ROMs test and ~100 µmol/L Fe^2+^ equivalents for the BAP test, as reported by the manufacturer. Both assays are CE-marked, validated for clinical research use, and demonstrate good reproducibility and accuracy as reported by the manufacturer They provide validated and reproducible indices of systemic oxidative stress, but they do not identify specific ROS and may be influenced by plasma constituents such as uric acid (for BAP) or ceruloplasmin activity (for d-ROMs), which should be considered when interpreting results.

### 2.4. Patient Demographics and Laboratory Measures

At enrollment, we recorded maternal age, gravidity, parity, fasting glucose, glycated hemoglobin (HbA1c), erythrocyte sedimentation rate (ESR), C-reactive protein (CRP), and bilirubin levels (total and direct). Pregnancy-related maternal comorbidities included anemia, arterial hypertension, infection, gestational weight gain, thrombophilia, hypothyroidism, autoimmune thyroiditis, polyhydramnios, and oligohydramnios. Maternal infections recorded during pregnancy were pregnancy-related, in most cases, asymptomatic bacteriuria or mild systemic infections. Maternal outcomes were defined according to standard obstetric criteria (Table S1). Anemia was defined as hemoglobin < 11 g/dL in the third trimester; hypertensive disorders (gestational hypertension, preeclampsia) were defined per ACOG guidelines [26]; Gestational weight gain was calculated as the difference between maternal weight at delivery and pre-pregnancy weight (information taken from clinical records). Classification of weight gain followed the Institute of Medicine (IOM) guidelines, which recommend total gestational weight gain of 11.5–16 kg for women with normal BMI (18.5–24.9 kg/m^2^), 7–11.5 kg for overweight women (BMI 25–29.9 kg/m^2^), and 5–9 kg for obese women (BMI ≥ 30 kg/m^2^) [27]. Amniotic fluid disorders were defined by ultrasound thresholds. Infections, thyroid disorders, and thrombophilia were recorded when clinically or laboratory confirmed. Neonatal outcomes were defined using institutional reference standards, represented in Table S1.

### 2.5. Statistical Analysis

Continuous variables are presented as mean ± standard deviation (SD) when normally distributed and as median with interquartile range (IQR) when distributions were skewed. Normality of data distributions was assessed using the Shapiro–Wilk test, and homogeneity of variances was evaluated using Levene’s test. Comparisons across compartments (maternal vs. cord vs. neonatal) were analyzed using repeated-measures ANOVA. Between-group differences (GDM vs. controls) were assessed by Welch’s *t*-test for continuous data, the Mann–Whitney U test for non-normally distributed variables, and Fisher’s exact test for categorical outcomes. Correlations were evaluated by Pearson’s coefficient. To assess the influence of glycemic control and maternal adiposity, subgroup analyses were conducted stratifying women with GDM by HbA1c (<6.5% vs. ≥6.5%) and by BMI (<30 vs. ≥30 kg/m^2^). Multivariate linear regression models were applied to evaluate whether differences in oxidative stress markers were independent of potential confounders. Maternal d-ROMs and BAP were used as dependent variables. Independent variables included GDM status (primary predictor), while maternal age, BMI, and HbA1c were considered potential confounders; BMI and HbA1c were additionally examined as possible effect modifiers. Multicollinearity between predictors was assessed using variance inflation factors, with all values <2, indicating no collinearity concerns. Regression coefficients (β) with 95% confidence intervals (CI) and *p*-values were reported. A two-sided *p*-value < 0.05 was considered statistically significant. All analyses were performed in SPSS v26.0 (IBM Corp, Armonk, NY, USA).

## 3. Results

### 3.1. Patient Demographics and Laboratory Data

**Figure 1:**
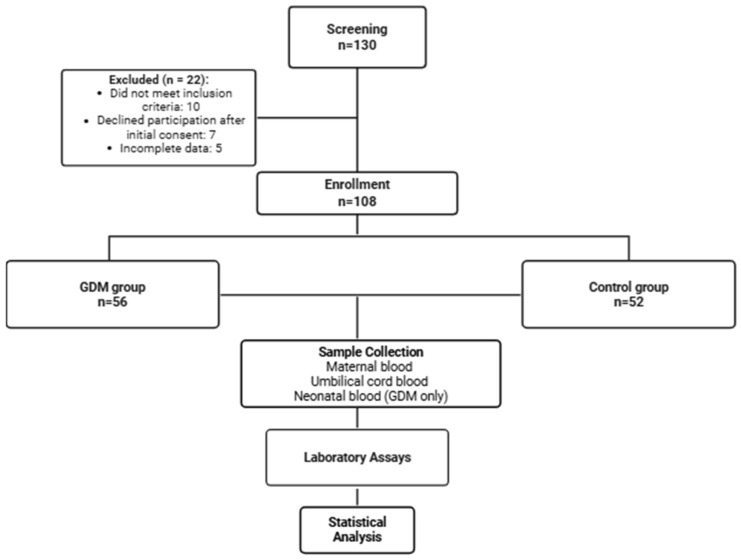
Flowchart illustrating participant recruitment, group allocation, and study workflow.

A total of 108 pregnant women were enrolled, including 56 with GDM and 52 normoglycemic controls. The recruitment process, exclusions, group allocation, and study workflow are summarized in a flowchart (Figure 1). Demographic and laboratory characteristics are represented in Table 1. Women with GDM were, on average, one year older than non-diabetic controls (31.16 ± 5.42 vs. 30.4 ± 4.38 years; *p* = 0.42). As expected, their fasting glucose was substantially higher (6.53 ± 1.85 vs. 4.23 ± 0.79 mmol/L; *p* < 0.001), and HbA1c had also higher levels (5.44 ± 0.73% vs. 4.67 ± 0.15%; *p* < 0.001). Inflammatory markers were also elevated in the diabetic group, with ESR at 42 (28–58) vs. 30 (18–46) mm/h (*p* < 0.001) and CRP at 2.3 (0.9–5.6) vs. 0.6 (0.3–1.2) mg/dL (*p* < 0.001).

### 3.2. Results of d-ROM in Different Sample Compartments

**Figure 2:**
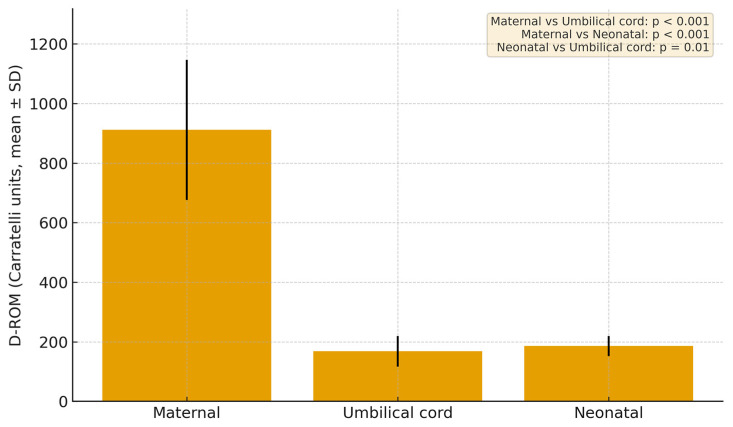
Mean Plasma Reactive Oxygen Metabolites in the group with GDM. *p*-values were calculated using paired *t*-tests.

D-ROM and BAP levels were evaluated in all pregnancies (*n* = 108). Maternal blood showed a mean of 911.2 ± 234.8 Carratelli units, umbilical cord blood 168.2 ± 51.5 Carratelli units, and neonatal blood 185.8 ± 33.5 Carratelli units (Figure 2). We performed paired *t*-tests on d-ROM values between maternal blood, umbilical-cord blood, and neonatal blood. The mean difference between maternal and neonatal blood was 778.7 Carratelli units (*p* < 0.001), indicating a highly significant elevation in maternal oxidative stress. Maternal levels likewise exceeded neonatal levels by 726.8 Carratelli units (*p* < 0.001). A weaker t but still statistically significant difference was found between neonatal and umbilical cord blood, with neonatal samples higher by 16.9 Carratelli units (*p* = 0.01).

### 3.3. Results of BAP in Different Sample Compartments

**Figure 3:**
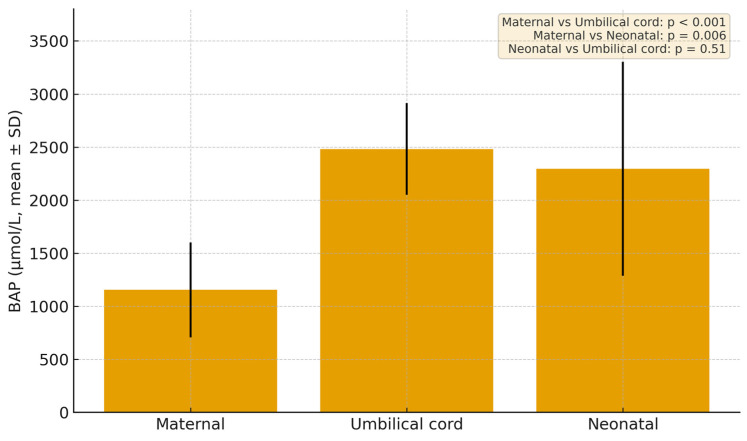
Mean Biological Antioxidant Potential in the group with GDM. *p*-values were calculated using paired *t*-tests.

For the BAP panel in the group with GDM, the results showed a maternal blood mean of 1154.73 µmol/L (SD = 447.02 µmol/L), an umbilical-cord mean of 2482.19 µmol/L (SD = 432.12 µmol/L), and a neonatal blood mean of 2296.16 µmol/L (SD = 1007.40 µmol/L) (Figure 3). Paired comparisons revealed that both cord and neonatal BAP values were significantly higher than maternal levels, with mean differences of –1338.8 µmol/L (*p* < 0.001) and –1123.6 µmol/L (*p* = 0.006), respectively, while the modest difference between neonatal and cord blood (−173.5 µmol/L) did not reach statistical significance (*p* = 0.51).

### 3.4. Comparison of d-ROM Between Groups

To evaluate whether GDM is associated with markers of elevated oxidative stress, we compared d-ROM measurements in maternal and umbilical-cord blood between our diabetes cohort and the control group.

**Figure 4:**
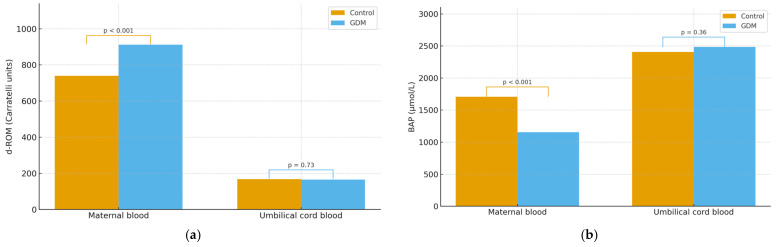
(**a**) Comparison of Plasma d-ROM Levels and (**b**) BAP Levels in GDM versus Control Group. *p*-values were obtained using independent two-sample *t*-tests with Welch’s correction.

In maternal blood, the diabetes group had a mean d-ROM of 911.23 Carratelli units, compared with 739.2 Carratelli units in controls; this difference was statistically significant (*p* = <0.001), indicating higher systemic oxidative stress in women with GDM. In contrast, umbilical cord blood d-ROMs averaged 165.2 Carratelli units in the group with GDM and 168.5 in controls, a difference that did not reach significance (*p* = 0.73) (Figure 4a). Because neonatal blood samples were only collected in the diabetes group, we were unable to compare neonatal-compartment oxidative stress between groups.

### 3.5. Comparison of BAP Between Groups

We compared BAP levels between women with GDM and non-diabetic controls using Welch’s *t*-test. In maternal blood, antioxidant capacity was markedly lower in the diabetes group (1154.73 vs. 1706.6 µmol/L; *p* < 0.001). In contrast, umbilical-cord BAP values were similar between groups (2482.19 vs. 2406.3 µmol/L; *p* = 0.36) (Figure 4b).

### 3.6. Correlations BAP/d-ROM

**Figure 5:**
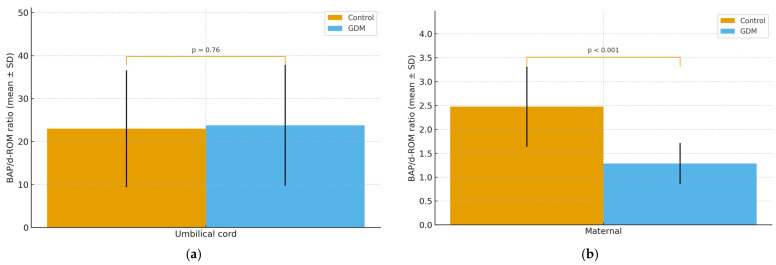
(**a**) Umbilical-Cord BAP/d-ROM Ratio and (**b**) Maternal-Blood BAP/d-ROM Ratio in GDM versus Control Group. *p*-values were obtained using independent two-sample *t*-tests with Welch’s correction.

We next calculated the BAP/d-ROM ratio in each compartment to calculate antioxidant capacity against oxidative burden. In umbilical-cord samples, the ratio was 23.78 ± 14.08 in the group with GDM versus 22.98 ± 13.60 in controls, a difference that did not reach statistical significance (*p* = 0.76) (Figure 5a). In the GDM group, the maternal-blood ratio averaged 1.283 ± 0.43, compared with 2.474 ± 0.84 in non-diabetic controls, a significant reduction in maternal antioxidant buffering (*p* < 0.001) (Figure 5b). Neonatal samples were only available for the diabetes group (mean ratio 14.30 ± 6.45), so no direct comparison to controls could be made.

### 3.7. Regression Analyses

To evaluate whether group differences persisted after adjustment for potential confounders, we performed multivariate linear regression analyses with maternal d-ROMs and BAP as dependent variables.

After adjustment for maternal age, BMI, and HbA1c, GDM remained a significant independent predictor of higher d-ROMs (β = +138.6 Carratelli units, 95% CI 64.5–212.7, *p* < 0.001). Similarly, GDM was independently associated with lower maternal BAP values (β = −395.4 µmol/L, 95% CI −602.2 to −188.6, *p* < 0.001).

### 3.8. Glycemic Control and Maternal BMI

**Figure 6:**
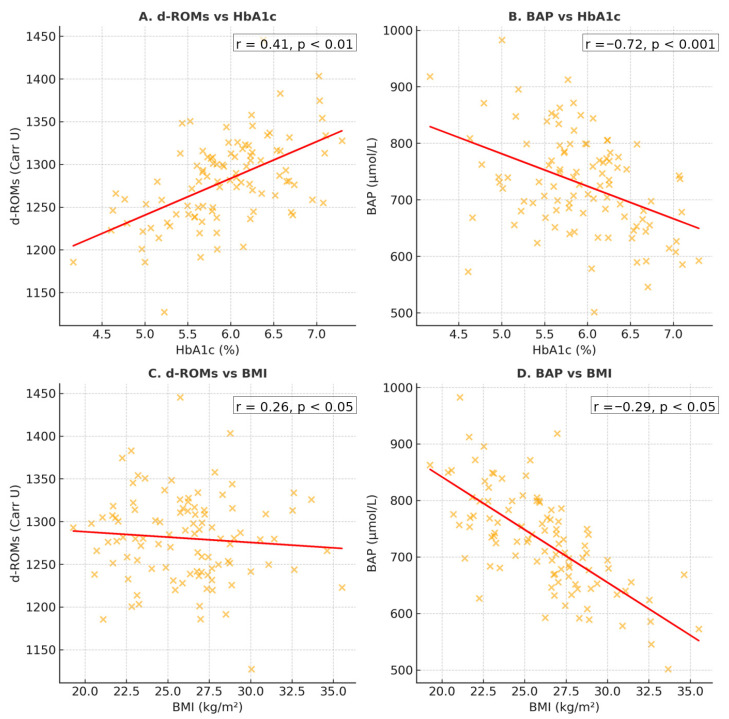
Scatter plots showing correlations of oxidative stress markers with clinical parameters. (**A**) D-Roms vs. Hba1c; (**B**) BAP Vs. Hba1c; (**C**) D-Roms Vs. BMI; (**D**) BAP Vs. BMI. Linear regression lines with 95% CI are shown. Correlation coefficients (*r*) and *p* values were obtained using Pearson correlation.

Correlation analyses revealed significant associations between oxidative stress parameters and maternal clinical variables (Figure 6). Maternal d-ROMs increased with rising HbA1c (*r* = 0.41, *p* < 0.01), while BAP values decreased as HbA1c increased *r* = −0.72, *p* < 0.001). In addition, higher maternal BMI at sampling correlated with elevated d-ROMs (*r* = 0.26, *p* < 0.05) and with lower BAP values (*r* = −0.29, *p* < 0.05).

To assess the effect of glycemic control, women with GDM were stratified according to HbA1c levels (<6.5% vs. ≥6.5%). Of the 56 women, 42 (75%) had HbA1c <6.5%, while 14 (25%) had HbA1c ≥6.5%. Maternal d-ROMs were higher in women with HbA1c ≥6.5% compared with those below this threshold (1020 ± 240 vs. 880 ± 225 Carratelli units, *p* = 0.04). Maternal BAP tended to be lower in the poorly controlled group (1050 ± 430 vs. 1180 ± 445 µmol/L, *p* = 0.12), and the resulting BAP/d-ROM ratio was significantly reduced (1.08 ± 0.42 vs. 1.35 ± 0.44, *p* = 0.03). Umbilical cord and neonatal d-ROMs and BAP values did not differ significantly between HbA1c subgroups.

Similarly, stratification by maternal BMI (<30 vs. ≥30 kg/m^2^) showed that women with a BMI ≥ 30 had slightly higher d-ROMs (960 ± 235 vs. 905 ± 220 Carratelli units, *p* = 0.09) and lower BAP values (1085 ± 410 vs. 1160 ± 455 µmol/L, *p* = 0.15), leading to a modestly reduced BAP/d-ROM ratio (1.13 ± 0.40 vs. 1.26 ± 0.46, *p* = 0.11). Umbilical cord and neonatal oxidative stress markers were not influenced by maternal BMI category.

### 3.9. Clinical Correlations

#### 3.9.1. Pregnancy-Related Complications

Finally, the pregnancy background and obstetric and fetal outcomes of the GDM and control groups are discussed. Of the 52 non-diabetic control subjects, comorbidity prevalence was as follows: 24 women had anemia, 9 had hypertensive disorders of pregnancy (HTAIS), 8 had documented infections, and 4 cases each of excessive gestational weight gain, thrombophilia, hypothyroidism, and oligohydramnios; no subjects exhibited autoimmune thyroiditis or polyhydramnios. Table 2 summarizes the mean and SD values between patients with and without the pregnancy-related complication in the control group.

In the 56 women with GDM, the following comorbidities were observed: 29 had anemia, 16 had HTAIS, 13 had infections, 8 had excessive gestational weight gain, 3 had oligohydramnios, and there were 2 cases each of thrombophilia, hypothyroidism, autoimmune thyroiditis, and polyhydramnios. Table 3 summarizes the mean and SD values between patients with and without pregnancy-related complications in the GDM group.

#### 3.9.2. Fetal Outcomes

In the non-diabetic control cohort of 52 mother–fetus pairs, we examined both maternal and umbilical-cord oxidative-stress markers (d-ROMs and BAP) in relation to neonatal outcomes, birth weight, and macrosomia, anemia, infection, hypoxia, respiratory distress, and intrauterine growth restriction. Among the 52 newborns, 9 were anemic, 6 were diagnosed with intrauterine growth restriction, 5 had an infection, 4 experienced intrauterine hypoxia, and 2 were diagnosed with macrosomia and respiratory distress. there were no cases of hypoglycemia, hypocalcemia, or hypomagnesemia. Maternal d-ROM values showed no linear relationship with neonatal weight (*p* = 0.86), nor did maternal BAP (*p* = 0.78). Similarly, cord-blood d-ROMs and BAP were not significantly correlated with weight (cord d-ROM: *p* = 0.42; cord BAP: *p* = 0.58). Nine infants were anemic, while mothers of the nine anemic babies had, paradoxically, substantially lower oxidative stress (mean d-ROM 533.2 CARR U Vs. 774.7 CARR U in non-anemic; *p* = 0.01) and higher antioxidant capacity (mean BAP 2074.4 Μmol/L Vs. 1734.3 µmol/L; *p* = 0.03).

Among the 56 newborns of diabetic mothers, there were 16 cases of intrauterine hypoxia, 12 of respiratory distress, 9 of anemia, 8 of each macrosomia, hypoglycemia, and infection, 6 of hypocalcemia, 6 of hypomagnesemia, and 5 cases of intrauterine growth restriction. We first examined the relationship between oxidative stress and birth weight. Maternal d-ROM values showed no significant linear association with neonatal weight (*p* = 0.77), nor did umbilical-cord d-ROM (*p* = 0.63). Next, we compared d-ROM levels by neonatal anemia status. We also examined neonates with macrosomia and found no significant differences in d-ROMs. Maternal BAP did not correlate with fetal weight (*p* = 0.8), and cord-blood BAP likewise showed no relationship (*p* = 0.91). Mothers whose infants experienced intrauterine hypoxia had modestly higher d-ROM and BAP levels than mothers of non-hypoxic infants, but neither difference reached statistical significance (d-ROM: *p* ≈ 0.43; BAP: *p* ≈ 0.2). Similarly, newborns who experienced respiratory distress did not exhibit any significant differences in oxidative-stress or antioxidant markers: neither maternal nor cord or fetal d-ROM and BAP levels differed between infants with or without distress (all *p* > 0.1). Overall, we did not observe any significant associations between oxidative-stress markers and adverse neonatal outcomes.

## 4. Discussion

Our study showed that women with GDM were older (although non-significant), exhibited higher fasting glucose, as expected, and a trend toward elevated inflammatory markers compared to normoglycemic controls. In the group with GDM, oxidative stress, measured by d-ROMs, was markedly greater in maternal blood than in paired umbilical-cord or neonatal samples, while BAP was lowest in maternal samples and progressively higher in cord and neonatal compartments. When compared to controls, diabetic mothers showed significantly increased systemic oxidative stress and depleted antioxidant reserves, whereas cord blood values remained comparable between groups. Consequently, the maternal BAP/d-ROM ratio was substantially reduced in GDM, indicating impaired redox balance, whereas the corresponding cord ratio did not differ. Finally, neither maternal nor cord redox indices correlated meaningfully with neonatal weight or most perinatal outcomes. Our results are consistent with earlier studies, further corroborating the body of evidence on oxidative stress alterations in diabetic pregnancies [28,29,30].

Research showed that, compared to normoglycemic pregnancies, women with GDM experienced higher rates of dystocia, cesarean delivery, abnormal amniotic fluid, premature membrane rupture, and other obstetric complications (*p* < 0.01). Their infants also faced more frequent fetal distress, macrosomia, small-for-gestational-age status, and prematurity (all *p* < 0.01) [31]. In our cohort, we did not observe statistically significant associations between maternal oxidative stress markers and any of the adverse maternal or neonatal outcomes.

Oxidative stress during pregnancy reflects an important relationship between ROS generation, driven by the placenta’s high mitochondrial density and abundant iron stores, and the maternal antioxidant defense network. Although some reports describe rising lipid peroxide levels in the second trimester and up-regulated extracellular antioxidants with advancing gestation [32], other findings indicate that the balance of total oxidant and total antioxidant status remains relatively constant from the first trimester through term [33]. When antioxidant systems adequately neutralize these pro-oxidant forces, placental and vascular function are preserved; however, failure of this redox homeostasis can precipitate adverse outcomes such as miscarriage, intrauterine growth restriction, preeclampsia, and vascular dysfunction [34,35]. In particular, elevated oxidative stress in late gestation associates with increased arterial stiffness and impaired endothelial reactivity [36,37].

Changes in oxidative stress markers have also been demonstrated in a variety of other pregnancy-related complications. In a scoping review, Zheng et al. [38] found that women who experience marked oxidative stress during pregnancy face an increased likelihood of developing pregnancy-induced hypertension, suffering postpartum hemorrhage, delivering infants with lower birth weights, and having neonates at greater risk for hyperbilirubinemia, fetal growth restriction, and birth asphyxia. Their findings also suggest that strict glycemic control before conception and throughout gestation, combined with targeted antioxidant therapies, may offer significant benefits for those at high risk of GDM [38].

Kawashiro et al. [39] measured d-ROMs and BAP in 126 pregnant women versus seven non-pregnant controls. At 36–37 weeks, d-ROMs rose significantly versus control and earlier trimesters, while BAP dropped markedly compared to every other group [39]. In a cross-sectional study, women with GDM showed consistently higher oxidative-stress markers than normoglycemic controls—not only later in pregnancy, but as early as the first trimester and still three months postpartum. The total oxidative status/total antioxidative status ratio was significantly elevated in the GDM group (*p* < 0.01), indicating a durable redox imbalance. Within the group with GDM, higher oxidative stress tracked with worse glycemic control (higher HbA1c), greater inflammation, and trends toward adverse neonatal outcomes, suggesting that a simple total oxidant status/total antioxidant status measurement could both flag early GDM risk and stratify disease severity [40]. Research using maternal and cord blood samples highlighted that, compared with non-diabetic controls, diabetic mothers and their infants exhibited persistently elevated markers of oxidative damage and diminished antioxidant status both before and after delivery [41]. Even more, amniotic fluid collected from pregnant women revealed that markers of oxidative damage were significantly elevated in preeclampsia, GDM, and infection compared with healthy pregnancies, whereas overall antioxidant enzyme activities were largely unchanged except for a selective up-regulation in the GDM subgroup [42]. There is a very small number of studies that have explored whether alterations in oxidative-stress biomarkers during early gestation can predict the later development of GDM, leaving a critical gap in our understanding of redox dynamics as a potential early warning signal for GDM [21]. Qiu et al. reported that elevated maternal urinary 8-OHdG in early pregnancy predicted later GDM risk [43], and, more recently, Gerszi et al. found that first-trimester serum total antioxidant capacity was independently increased in women who went on to develop GDM, suggesting a compensatory rise in antioxidants in response to elevated free radicals [44]. Murthy et al. [45] showed that women with GDM displayed a clear pro-inflammatory, pro-oxidant profile compared with normoglycemic controls. Serum uric acid was higher, as were the cytokines tumoral necrosis factor (TNF)-α, interleukin (IL)-6, and IL-8, whereas key antioxidant enzymes, superoxide dismutase (SOD) and glutathione transferase (GST), were significantly lower [45]. Before delivery, preeclamptic women exhibited the highest anti-angiogenic and oxidative-stress markers and the lowest pro-angiogenic and antioxidant indices, with values in gestational hypertension falling between preeclampsia and normotensive pregnancies [46].

Our findings of elevated maternal oxidative stress in GDM are consistent with recent evidence that redox imbalance may contribute to placental inflammatory signaling. A recent study found that high-mobility group box 1 (HMGB1) expression was considerably higher in placental tissue from GDM pregnancies, although vascular cell adhesion molecule-1 (VCAM-1) levels were not significantly different between groups. HMGB1, a redox-sensitive damage-associated molecular pattern (DAMP), plays an important role in immune activation and is known to respond to oxidative stressors. Its upregulation in GDM placentas may be a direct consequence of the systemic rise in ROS reported in our group. The study’s Gene Ontology analysis identified functional linkages between HMGB1 and immune-related biological processes, specifically immune system activation [47]. These findings imply that maternal redox status may not only act as a systemic marker but also promote local placental inflammation. Therapeutically, HMGB1 inhibition and modification of its receptor signaling may provide new options for reducing inflammation in GDM. Lifestyle modification remains the primary strategy for GDM management and has been shown to reduce macrosomia, shoulder dystocia and preeclampsia, but a subset of women still require insulin or oral agents to achieve glycemic control, and while insulin does not cross the placenta, metformin and glyburide do, with uncertain long-term safety for offspring and notable treatment-failure rates for both drugs [48,49,50]. Interventions such as high-dose vitamin C and E co-supplementation, myo-inositol, omega-3 fatty acids, and probiotics have all been evaluated for their potential to enhance antioxidant capacity and attenuate oxidative stress during pregnancy [51]. Maged Et Al. also investigated the role of antioxidants regarding fetal outcomes [52]. Recent randomized trials have demonstrated that adjunctive probiotic supplementation (***Lactobacillus acidophilus*** LA-5, ***Bifidobacterium bifidum*** BB-12, ***Streptococcus thermophilus*** STY-31, ***Lactobacillus delbrueckii bulgaricus*** LBY-27) can lower high-sensitivity CRP, TNF-α, glutathione peroxidase (GPx), and malondialdehyde (MDA) while boosting glutathione reductase in GDM patients [53,54]. Furthermore, co-supplementation with magnesium, zinc, calcium, and vitamin D further reduces hs-CRP and MDA and raises total antioxidant capacity (TAC), with associated reductions in neonatal macrosomia [55]. However, these studies were limited by short intervention durations, the lack of healthy-pregnancy controls, and the absence of long-term outcome data, underscoring the need for larger, longer-term trials and prevention-focused research.

## 5. Strengths, Limitations, and Perspectives

This study has multiple strengths that contribute to our understanding of redox status in GDM. We provide an expanded view of redox dynamics at the maternal-fetal interface by simultaneously analyzing oxidative stress and antioxidant defense markers in maternal, neonatal, and cord compartments. The utilization of proven commercial assays (d-ROMs and BAP tests) contributes to the accuracy and reproducibility of our measurements. Correlating biomarker levels with newborn measures provides a preliminary understanding of the physiological effects of maternal oxidative stress. However, limitations must also be acknowledged. This was a cross-sectional observational study; thus, drawing conclusions about causality between oxidative stress and pregnancy outcomes is not feasible. A limitation of our methodology is that the d-ROMs and BAP assays provide global indices of oxidative stress and antioxidant capacity rather than quantifying specific reactive oxygen species. The BAP test is strongly influenced by uric acid and other plasma antioxidants, while the d-ROMs signal may be affected by ceruloplasmin activity in addition to hydroperoxide levels. Most importantly, the study’s relatively small sample size may have hampered the detection of more subtle relationships or subgroup differences. This limitation reduces the strength of our conclusions. Subgroup analyses according to pregnancy-related complications should be interpreted with caution, as the sample sizes were small and the study was not specifically powered for these comparisons. These results are therefore exploratory and hypothesis-generating rather than confirmatory. Finally, no long-term neonatal follow-up was performed, limiting inferences about the persistence of redox-related effects beyond delivery. These discoveries have various practical applications in clinical care and future study. The increase in maternal oxidative stress supports the potential use of redox biomarkers in risk stratification models for adverse maternal and newborn outcomes. Such biomarkers could supplement established diagnostic criteria and guide personalized interventions, such as antioxidant-based therapy. However, before clinical trials begin, larger longitudinal investigations are required to evaluate whether reducing oxidative stress improves prenatal and postnatal outcomes. In terms of generalizability, the study sample was drawn from a single center population, which may restrict the external validity of the findings. Future multicenter research in heterogeneous populations will be required to establish the reproducibility and general applicability of these findings.

## 6. Conclusions

This cross-sectional analysis suggests a potential redox imbalance in GDM, as reflected by increased d-ROMs and decreased BAP levels. while the umbilical-cord and neonatal compartments preserve relatively higher antioxidant capacity. Compared to normoglycemic controls, women with GDM exhibited significantly greater systemic oxidative stress without a corresponding increase in neonatal oxidative markers, and the maternal BAP/d-ROM ratio was reduced. The lower maternal BAP/d-ROM ratio may serve as a potential biomarker of oxidative burden in GDM, although this requires further validation. Overall, the results suggest that monitoring maternal oxidative status during pregnancy could provide useful insights, but conclusions remain preliminary. The limited sample size restricts the statistical power and generalizability of our results. However, these findings should be interpreted as preliminary and hypothesis-generating; further studies with larger cohorts are essential to validate these observations.

## Figures and Tables

**Table 1:** Demographic and laboratory data.

Parameter	Gestational Diabetes Group	Control Group	*p*-Value
Age (years) mean ± SD	31.16 ± 5.42	30.4 ± 4.38	0.42
BMI (kg/m^2^) mean ± SD	28.7 ± 4.5	25.1 ± 3.8	<0.001
Gestational age at birth (weeks) mean ± SD	38.23 ±2.47	38.67 ±0.63	0.20
Gestational age at maternal sampling (weeks) mean ± SD	33.21 ±0.70	34.1 ±0.35	<0.001
Glucose (mmol/L) mean ± SD	6.53 ± 1.85	4.23 ± 0.79	<0.001
HbA1c (%) median (IQR)	5.44 ± 0.73	4.67 ± 0.15	<0.001
ESR (mm/h) median (IQR)	42 (28–58)	30 (18–46)	<0.001
CRP (mg/dL) median (IQR)	2.3 (0.9–5.6)	0.6 (0.3–1.2)	<0.001
Total Bilirubin (µmol/L) median (IQR)	9.0 (7.5–11.2)	8.7 (6.8–10.1)	0.39
Direct Bilirubin (µmol/L) median (IQR)	3.9 (2.5–6.0)	4.1 (3.2–5.0)	0.98

Abbreviations: BMI—body mass index; CRP—C-reactive protein; ESR—erythrocyte sedimentation rate; HbA1c—glycated hemoglobin; IQR—interquartile range.

**Table 2:** Maternal oxidative stress (d-ROMs) and antioxidant capacity (BAP) according to pregnancy-related complications in the non-diabetic control group. Mean and standard deviation values are presented for each subgroup.

Complication	Number of Patients	d-ROMs (Mean and SD)	*p*-Value	BAP (Mean and SD)	*p*-Value
Anemia	Yes (*n* = 24)	748.7± 123.7	0.91	1 586 ± 3 558.2	0.90
	No (*n* = 28)	743.9 ± 192.4		1 679.3 ± 281.4	
HTAIS	Yes (*n* = 9)	858.5 ± 142.9	0.03	1 748.7 ± 139.2	0.46
	No (*n* = 43)	725.7 ± 147.9		1 683.9 ± 489.8	
Infection	Yes (*n* = 8)	675.5 ± 261.3	0.38	1 915.3 ± 317.0	0.06
	No (*n* = 44)	763.5 ± 139.9		1 648.7 ± 465.0	

Abbreviations: BAP—biological antioxidant potentialantioxidant; d-ROMs—derivatives of reactive oxygen metabolitesoxidative; HTAIS—hypertensive disorders of pregnancy; *n*—number; SD—standard deviation.

**Table 3:** Maternal oxidative stress (d-ROMs) and antioxidant capacity (BAP) according to pregnancy-related complications in the diabetic control group. Mean and standard deviation values are presented for each subgroup.

Complication	Number of Patients	d-ROMs (Mean and SD)	*p*-Value	BAP (Mean and SD)	*p*-Value
Anemia	Yes (*n* = 29)	912.3 ± 232.7	0.09	1076.6 ± 43.5	0.07
	No (*n* = 27)	832.1 ± 95.2		1265.7 ± 525.5	
HTAIS	Yes (*n* = 16)	854.9 ± 164.9	0.23	906.1 ± 274.7	<0.001
	No (*n* = 40)	928.9 ± 281.2		1243.6 ± 411.5	
Infection	Yes (*n* = 13)	883.6 ± 147.7	0.84	1234.1 ± 339.7	0.34
	No (*n* = 43)	895.8 ± 278.8		1122.8 ± 428.3	
Excessive gestational weight gain	Yes (*n* = 8)	977.4 ± 175.6	0.18	1030.6 ± 197.6	0.20
	No (*n* = 48)	874.3 ± 258.1		1153.8 ± 419.7	
Oligohydramnios	Yes (*n* = 3)	733.17 ± 96.27	0.03	812.5 ± 163.05	0.01
	No (*n* = 53)	946.24 ± 251.90		1324.27 ± 449.95	

Abbreviations: HTAIS—hypertensive disorders of pregnancy.

## Data Availability

Data is available on request from the corresponding author.

## Supplementary Materials

The following supporting information can be downloaded at: https://www.mdpi.com/article/10.3390/jcm14197003/s1. Table S1: Definitions of study variables.
