# Supplementary material for: Assessment of Maternal–Fetal Redox Balance in Gestational Diabetes Mellitus: A Cross-Sectional Study
Source: J Clin Med. 2025 Oct 3;14(19):7003. doi: 10.3390/jcm14197003 (PMC12524906; doi:10.3390/jcm14197003)
Supplement: Supplementary file 1 [file jcm-14-07003-s001.zip › jcm-3854151-supplementary.pdf]

Table S1. Definitions of study variables

Variable / Outcome	Definition / Diagnostic Criteria	Coding
**Anemia (maternal)**	Hemoglobin <11.0 g/dL in 3rd trimester (or hematocrit <33%)	Yes / No
**Gestational hypertension**	BP ≥140/90 mmHg on ≥2 occasions ≥4 h apart, without proteinuria	Yes / No
**Preeclampsia**	BP ≥140/90 mmHg after 20 weeks plus proteinuria (≥300 mg/24 h or protein/creatinine ≥0.3) or end-organ dysfunction	Yes / No
**Infection (maternal)**	Clinically or microbiologically documented infection during pregnancy	Yes / No
**Excessive gestational weight gain**	Weight gain above 2009 Institute of Medicine (IOM) recommendations by pre-pregnancy BMI category	Yes / No
**Thrombophilia**	Documented inherited or acquired thrombophilia	Yes / No
**Hypothyroidism**	Elevated TSH with low FT4 (pregnancy-adjusted) or use of levothyroxine	Yes / No
**Autoimmune thyroiditis**	Positive TPO and/or Tg antibodies with clinical diagnosis	Yes / No
**Polyhydramnios**	Amniotic fluid index ≥24 cm or deepest vertical pocket ≥8 cm	Yes / No
**Oligohydramnios**	Amniotic fluid index ≤5 cm or deepest vertical pocket <2 cm	Yes / No
**Neonatal anemia**	Hemoglobin <13 g/dL and/or hematocrit <45% within the first 28 days of life	Yes / No
**Neonatal respiratory distress**	Clinical diagnosis requiring supplemental O_2, CPAP, or mechanical ventilation	Yes / No

**Variable / Outcome**	**Definition / Diagnostic Criteria**	**Coding**
**Neonatal infection**	Culture-proven or clinician-diagnosed early-onset sepsis (<72 h of life)	Yes / No
**Birth weight**	Recorded in delivery records; classified as continuous (grams) and categorical (low birth weight <2500 g; macrosomia >4000 g)	Continuous / Binary
